# Developmental Dyscalculia and Automatic Magnitudes Processing: Investigating Interference Effects between Area and Perimeter

**DOI:** 10.3389/fpsyg.2017.02206

**Published:** 2017-12-21

**Authors:** Hili Eidlin-Levy, Orly Rubinsten

**Affiliations:** Edmond J. Safra Brain Research Center for the Study of Learning Disabilities, Department of Learning Disabilities, University of Haifa, Haifa, Israel

**Keywords:** developmental dyscalculia, magnitude processing, geometric processing Stroop task, inhibition processing

## Abstract

The relationship between numbers and other magnitudes has been extensively investigated in the scientific literature. Here, the objectives were to examine whether two continuous magnitudes, area and perimeter, are automatically processed and whether adults with developmental dyscalculia (DD) are deficient in their ability to automatically process one or both of these magnitudes. Fifty-seven students (30 with DD and 27 with typical development) performed a novel Stroop-like task requiring estimation of one aspect (area or perimeter) while ignoring the other. In order to track possible changes in automaticity due to practice, we measured performance after initial and continuous exposure to stimuli. Similar to previous findings, current results show a significant group × congruency interaction, evident beyond exposure level or magnitude type. That is, the DD group systematically showed larger Stroop effects. However, analysis of each exposure period showed that during initial exposure to stimuli the DD group showed larger Stroop effects in the perimeter and not in the area task. In contrast, during continuous exposure to stimuli no triple interaction was evident. It is concluded that both magnitudes are automatically processed. Nevertheless, individuals with DD are deficient in inhibiting irrelevant magnitude information in general and, specifically, struggle to inhibit salient area information after initial exposure to a perimeter comparison task. Accordingly, the findings support the assumption that DD involves a deficiency in multiple cognitive components, which include domain-specific and domain-general cognitive functions.

## Introduction

In the past few years, the relationship between numbers and other magnitudes, such as space and time, has evoked a great deal of interest (Walsh, 2003; Dehaene and Brannon, 2010; Gebuis and Reynvoet, 2012a; Newcombe, 2014; Leibovich et al., 2016; McCaskey et al., 2017). Walsh (2003) was the first to suggest the existence of a common processing mechanism for time, space, and quantity, and he established a Theory of Magnitude (ATOM). Evolutionarily, such a mechanism enabled simultaneous processing of numerical, temporal, and spatial features of the world in order to produce adaptive reactions. Following Walsh’s theory, a wealth of knowledge appeared with regards to magnitude and numerical associations (Pinel et al., 2004; Dakin et al., 2011; Ren et al., 2011; Stoianov and Zorzi, 2012; Tibber et al., 2012; Leibovich et al., 2016; McCaskey et al., 2017). Specifically, recent research implies the existence of a magnitude estimation mechanism representing both numerical and other continuous magnitudes, including area, perimeter, length, volume, time, etc. (Lourenco and Longo, 2010; Dakin et al., 2011; Gebuis and Reynvoet, 2012b; Leibovich and Henik, 2013, 2014; Newcombe, 2014; Leibovich et al., 2016; Lourenco and Bonny, 2016).

Accumulating evidence shows that magnitude information is probably more perceptually salient than numerical information, and thus may have developed earlier (Feigenson et al., 2002; Gebuis and Reynvoet, 2012a; Odic et al., 2012). As an example, infant research shows that continuous magnitudes, such as the contour length (perimeter) or size (area) of figures, are more salient to infants than the number of items (Clearfield and Mix, 1999; Feigenson et al., 2002). Despite the fact that continuous magnitude processing seems to be as important as numerical processing for daily performance (Gebuis and Reynvoet, 2012b; Leibovich and Henik, 2013, 2014), comparisons between different types of continuous magnitudes (e.g., area and perimeter) have received much less scientific attention.

The current study aims to deepen the knowledge about continuous magnitude processing and has three main purposes. The first purpose is to investigate automatic processing (namely, processed spontaneously and with no need for monitoring; Tzelgov, 1997) of two continuous magnitudes, area and perimeter. The second purpose is to explore general interactions between numerical cognition and magnitude processing by inclusion of a specific clinical population – participants with deficient numerical abilities (developmental dyscalculia or DD). The third purpose is to investigate whether the DD group benefits from continuous exposure to stimuli as much as the control group.

### Area and Perimeter Processing

As mentioned, the study aims to investigate the automaticity of area and perimeter magnitudes. The automaticity of magnitude processing is investigated here via a Stroop–like task. Generally, in Stroop tasks it is typically found that participants cannot ignore irrelevant dimensions of the task (e.g., the physical size of a digit), which are processed involuntarily and interfere with the processing of the relevant dimension (e.g., the actual quantity that the digit represents; Henik and Tzelgov, 1982). Here, we adopted a Stroop-like comparison task created by Babai et al. (2006), which compared the automaticity of area vs. perimeter processing. Previous findings demonstrated that in a perimeter comparison task, the figure’s area was processed involuntarily and thus affected perimeter judgment. In the *congruent condition*, the figure with the greater perimeter also had a larger area, while in the *incongruent condition*, the perimeters of the two figures were equal, while one of them had a larger area. Results indicated that participants showed a clear *Stroop effect*, namely they responded faster to congruent than to incongruent trials (Stavy et al., 2006; Stavy and Babai, 2008, 2010). Thus, the irrelevant area aspect might be automatically processed and, despite being irrelevant to the task, interfere with perimeter processing. Notably, the opposite task, that is when participants were asked to determine which figure has the largest area (and to ignore the irrelevant perimeter) was examined as well. Results showed that of the two tasks, area comparisons were significantly faster than perimeter comparisons. However, unlike the perimeter comparison task (where area is irrelevant to the task), in the area comparison task (where perimeter is irrelevant to the task) no differences were found between congruent and incongruent trials (Babai et al., 2006). The researchers inferred that area is more salient and more rapidly processed (i.e., more automatic) than perimeter.

To this end, it is not clear whether one magnitude (area) can be more perceptually salient than the other (perimeter). According to general magnitude mechanism theories, both magnitudes should be involuntarily processed and hence are supposed to interfere with each other to a similar degree (Newcombe, 2014; Leibovich et al., 2016). On the other hand one magnitude, in this case area (as found in Babai et al., 2006), can interfere with the processing of another magnitude (perimeter), but not vice versa. A possible solution for this contradiction is that magnitude saliency differs due to task demands (Spelke et al., 2010). Spelke et al. (2010) identified two core systems used in geometric processing: the first is important for navigation at large – scale surfaces (Cheng and Newcombe, 2005) while the second system is used for small object recognition, such as the figures in Babai et al.’s (2006) task. Each system relates to different neural and cognitive processing, with limited transference between them (Derdikman and Moser, 2010; Huang and Spelke, 2015). Accordingly, a particular magnitude can be automatically processed by one of the core systems but not by the other. For instance, the processing of a surface’s layout (or, in other words, *perimeter*) is highly crucial for successful navigation (Hermer and Spelke, 1994; Hermer-Vazquez et al., 1999; Lee et al., 2012) but not as crucial for recognition of 2D figures (Lee and Spelke, 2011).

### Developmental Dyscalculia and Magnitude Processing

The current research also aims to expand the knowledge about the interactions between numerical cognition and magnitude processing by including a specific clinical population – participants with deficient numerical abilities (DD). DD is a specific deficit of numerical and mathematical abilities, with a neuro-anatomical source (Rotzer et al., 2008; Kaufmann et al., 2013), affecting about 3.6–6.5% of the population (Geary, 1993; Shalev and Gross-Tsur, 2001; Butterworth, 2005; Reigosa-Crespo et al., 2012). Individuals with DD fail to master common numerical and arithmetical skills, such as numerical comparisons (Rubinsten and Henik, 2005; Mussolin et al., 2010a), arithmetical fact retrieval (Mazzocco et al., 2008), and procedural knowledge (Desoete et al., 2009). Currently, there is a debate about the cognitive mechanisms that underlie numerical difficulties, and DD seems to be a heterogeneous disorder (Rubinsten and Henik, 2009; Kaufmann et al., 2013; Träff et al., 2016). One main explanation for the heterogeneity of DD is that numerical ability cannot be described as a single cognitive mechanism and arithmetic competence relies on domain-specific as well as domain-general skills (Rubinsten and Sury, 2011; Kaufmann et al., 2013; Szűcs and Goswami, 2013; Bugden and Ansari, 2015; Huber et al., 2015; Noël et al., 2016). From one perspective, individuals with DD may have a domain-specific deficient “number sense,” manifested by difficulties with representing and manipulating all kinds of numerical notations: non-symbolic – such as dot arrays (Price et al., 2007; Mussolin et al., 2010b), or symbolic, namely Arabic numerals and number words (Rubinsten and Henik, 2005, 2006; Mussolin et al., 2010a). Concurrent with the general magnitude processing mechanism theory, they may also ineffectively process continuous magnitudes and have deficient “magnitude sense” (Leibovich et al., 2016). It is worth noticing though, that in a recent study adolescents with DD showed similar performance, on both behavioral and neuronal levels, as typical developing peers when performing non-symbolic numerical comparison tasks (McCaskey et al., 2017). However, participants with typical development activated domain-specific magnitude related areas while performing the task, while participants with DD activated domain-general frontal areas, related to inhibition and working memory. Accordingly, the authors inferred that domain–general deficits could also account for the development of DD.

Indeed, the role of inhibition, a domain–general mechanism, in intact and deficient numerical processing has also received scientific attention (Ashkenazi et al., 2009; Ashkenazi and Henik, 2010; Wang et al., 2012; Szucs et al., 2013a; Bugden and Ansari, 2015; Noël et al., 2016). From this perspective, individuals with DD show deficient performance on numerical tasks due to failure to inhibit irrelevant magnitude information, such as the overall area of dot arrays in a non-symbolic comparison task (Bugden and Ansari, 2015) or physical size in the numerical Stroop task (Szucs et al., 2013a). It is important to notice that these findings remained consistent after controlling for comorbidity (Wang et al., 2012).

### The Current Research

The discussion of a general magnitude processing mechanism as a basis for numerical cognition development seems to be more relevant than ever (Gebuis and Reynvoet, 2012b; Leibovich and Henik, 2014; Newcombe, 2014; Lourenco and Bonny, 2016; Leibovich et al., 2016). However, there exists no extensive work dealing with intact and deficient processing of this system (Skagerlund and Träff, 2014; McCaskey et al., 2017). Moreover, it is necessary to differentiate between “pure,” domain-specific, and domain-general mechanisms when performing magnitude comparison tasks, in order to define the source of DD difficulties.

Hence, we compared participants with DD and typically developing participants while performing a Stroop-like task (adopted from Babai et al., 2006). We expected to find group differences, as measured by Stoop effects (incongruent minus congruent trials) in both area and perimeter comparison tasks. According to domain-specific magnitude processing deficits (Newcombe, 2014; Leibovich et al., 2016), the DD group is expected to show smaller Stroop effects implying that they do not process the irrelevant magnitude (similar to the numerical Stroop task, Rubinsten and Henik, 2005). On the other hand, domain-general deficits (Wang et al., 2012; Szucs et al., 2013a; Bugden and Ansari, 2015) should result in larger Stroop effects in the DD group, due to a deficit in the ability to ignore irrelevant magnitude information.

Another interesting specific question is whether participants with DD will show similar deficient processing on both the area and perimeter tasks. If the area component is indeed more perceptually salient (Babai et al., 2006), the DD group should show larger Stroop effects on the perimeter task (in which area is irrelevant to the task), namely they should find it harder to ignore the irrelevant area aspect.

The cognitive method as well as the statistical analysis of the current study enabled us not only to study the differences between automatic processing of area vs. perimeter (i.e., investigating magnitude sense in DD), but also to investigate whether DD participants indeed perform poorly or differently on continuous magnitude processing tasks in initial vs. proficiency stages of learning (i.e., to investigate learning functions in DD). Earlier studies showed that even a small number of rehearsals of numerical problems led to automatic processing and to changes in brain functions (Ischebeck et al., 2007; Aubin et al., 2016). For instance, Ischebeck et al. (2007) found that very short training (eight repetitions) in multiplication problems led to a decrease in the activity of fronto-parietal brain areas related to calculation and numerical processing (Menon et al., 2000; Dehaene et al., 2003). On the other hand, the training also resulted in increased activity in temporo-parietal regions known to be involved in arithmetic fact retrieval (Dehaene et al., 2003). Recently, it was proposed that DDs’ deficits in inhibition of irrelevant numerical information can also represent difficulties with consolidating learned information and with performing the shift from initial computing based processing to automatic retrieval based processing (Ischebeck et al., 2007; Aubin et al., 2016). Indeed, Aubin et al. (2016) suggested that people with DD may be less able to consolidate a numerical task within the frontal-parietal region and must instead rely on their working memory. Consequently, there would be limited progression to recalling numerical information and a continued dependence on working memory. Accordingly, it is predicted here that people with DD may need more rehearsals in order to attain proficiency in performing magnitude tasks. Such difficulty with consolidating learned knowledge and with using advanced retrieval strategies will produce consistent group differences predicted to be prominent in the perimeter task, evident even after continuous exposure to stimuli.

## Materials and Methods

### Participants

Fifty-seven adults, 27 typically developing (i.e., control group; including 9 males, 18 females; mean age = 24.92 years, *SD* = 2.67 years) and 30 with DD (2 males, 28 females; mean age = 24.43 years, *SD* = 2.74 years) participated in the study. All participants were university students and had successfully completed math matriculation exams. Participants were recruited by advertisements distributed on campus and gave written consent to participate in the experiment. Some of them were paid about USD15 for their participation, while other received credit points for academic courses. The study was carried out in accordance with the recommendations of the ethics committee of the University of Haifa with written informed consent from all participants. All participants gave written informed consent in accordance with the Declaration of Helsinki. The protocol was approved by the ethics committee of the University of Haifa (No. 108/09).

### Classification and Assessment Criteria

Participants were assigned to either a control or a DD group, using the “Israeli learning function diagnosis system” (also titled “MATAL” in Hebrew) for high school and higher education students (National Institute for Testing and Evaluation). This diagnostic tool is composed of a set of standardized computerized tests and questionnaires intended for diagnosing learning disabilities in high school and higher education students. All tests and questionnaires used are nationally normalized. All participants performed numerical tasks (simple calculation and procedural knowledge calculation) and reading related tasks (text reading, phoneme omission, and rapid naming). They also answered a questionnaire (based on the DSM) regarding their attention ability, and performed Raven’s Standard Progressive Matrices (SPM) test (Raven et al., 1995) in order to rule out non-verbal mental disabilities.

Participants were defined as having DD if their scores in either RT or accuracy (ACC) measures on the simple calculation and procedural knowledge tests were worse than mean -1 SD and their scores in the reading and attention tests were more than +1 SD above the mean. Participants in the control group scored better than mean -1 SD in numerical, reading, and attention tests. All participants also scored above the 25th percentile in the SPM test. Independent *t*-tests were conducted on the different test results. The two groups differed significantly in both numerical tests (for mean test results and *p*-values of independent *t*-tests see **Table 1**). As significant group differences were also evident in reading related tasks, group results did not exceed 1SD lower than the norm score, hence it can be assumed that all participants were good readers.

**Table 1:** Mean standard scores and group differences in the screening tests of the two groups.

Test		Control		DD		*t*-test	
Mean scores		ACC	RT	ACC	RT	ACC	RT
Text reading		0.56	0.42	0.48	-0.31	0.37	2.78^∗∗^
Phoneme omission		0.46	0.65	-0.05	0.01	2.34^∗^	2.53^∗^
Rapid naming	Objects		0.14		-0.32		1.94
	Letters		0.64		0.33		1.21
	Numbers		0.42		-0.15		2.08^∗^
Questionnaire	Attention	0.09		0.12			-0.37
	Impulsiveness and hyperactivity	0.00		0.86			-1.57
Simple calculation		0.61	0.49	-1.3	-1.37	4.79^∗∗^	5.93^∗∗^
Procedural knowledge		0.46	0.59	-1.55	-1.26	6.05^∗∗^	6.92^∗∗^
SPM (percentiles)		58.4		52.43		1.25	

*ACC = standard scores for accuracy rates; RT = standard scores for reaction times; SPM = Standard Progressive Matrices (Raven et al., 1995); Sig. = independent *t*-test significance between control and DD groups. Significance values: ^∗^*p* < 0.05; ^∗∗^*p* < 0.01*.

### The Experimental Tasks – Area and Perimeter Tasks

The experiment was run on a PC using E-Prime 2.0 software, and contained two tasks, based on the comparison task of Babai et al. (2006). In each task, participants were requested to relate to a specific aspect of the stimuli (i.e., area or perimeter). Participants were presented with two polygons in each trial, and were asked to decide which polygon had the largest amount of one of the magnitude aspects (area/perimeter). Each block contained a similar number of congruent trials (where both area and perimeter are increased between stimuli) and incongruent trials (where one aspect is increased while the other is reduced between stimuli). Semi-neutral trials (where one aspect is equal and the other differs between stimuli) and equal trials (where both aspects are equal between stimuli) were added as fillers.

### Stimuli

**FIGURE 1:**
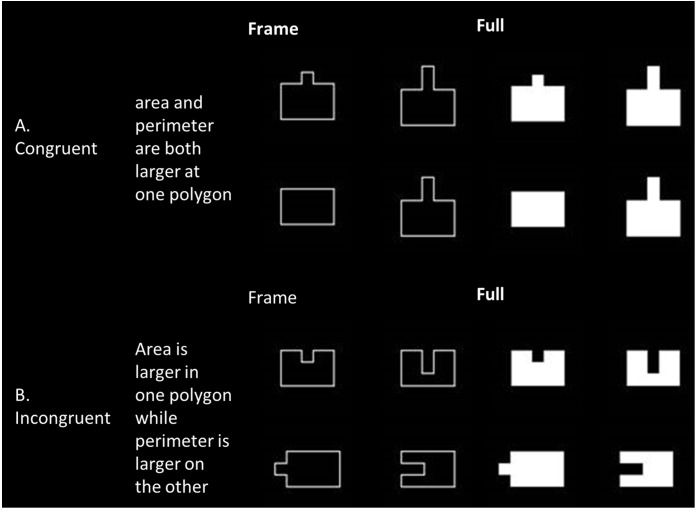
Examples of framed and filled stimuli, arranged by congruency. **(A)** Examples of congruent stimuli, where both area and perimeter are increased in both polygons. **(B)** Examples of incongruent stimuli, where a different aspect is increased in each polygon.

Each stimulus was composed of two figures – a basic rectangle or a polygon derived from the basic rectangle by adding or subtracting one or two squares (the size of each square was 1/12 of the basic rectangle size). Pairing of polygons was based on their protrusion direction (into the basic rectangle, see **Figure 1B**, or protruding out of it, see **Figure 1A**) and congruency rules (described above). Each polygon was ascribed to two different stimuli in order to avoid visual bias. Moreover, in order to avoid visual bias and learning, each stimulus was presented eight times, four times on the left side of the fixation point and four times on the right side of the fixation point. Each stimulus was also presented twice in four directions – two on the vertical axis – original (turning up) or rotated by 180° (turning down) and two on the horizontal axis – rotated by 90° to the left (turning left) or to the right (turning right).

**FIGURE 2:**
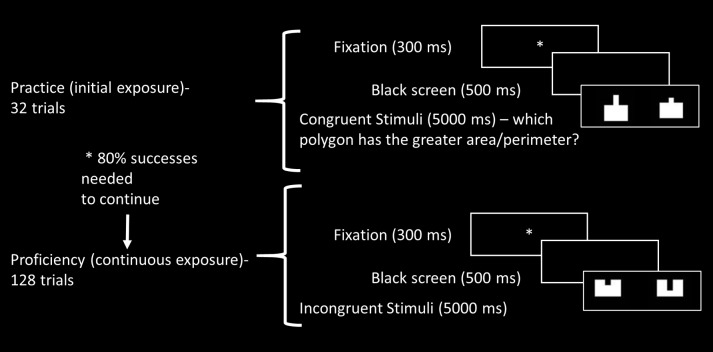
Illustration of experimental block, including initial (practice) and continuous exposure (proficiency) trials.

The task contained two blocks, one with filled figures and the other containing framed only figures, in order to avoid visual bias of one aspect (Stavy and Babai, 2008). Each block contained 32 “initial exposure” trials (following Ischebeck et al., 2007), representing the *practice* phase. We aimed to capture the reaction to unfamiliar stimuli – eight congruent trials (both area and perimeter were larger in one of the polygons), eight incongruent trials (where the area was larger for one polygon while the perimeter was larger in the other), and 16 filler trials (in which one or both aspects remained equal). After succeeding on more than 80% of the practice trials (in the first or second presentation), participants were able to continue to the continuous exposure, or *proficiency* phase, representing automatization levels. The proficiency phase contained 128 trials – 32 congruent trials (both area and perimeter were larger in one of the polygons), 32 incongruent trials (where the area was larger for one polygon while the perimeter was larger in the other), and 64 filler trials (in which one or both aspects remained equal). The filler trials enabled sufficient presentation of each stimulus and contained 32 equal trials, with the same polygons presented at different rotations, and 32 semi–neutral trials, where one aspect (area or perimeter) remained constant between polygons while the other differed. Since there were two experimental tasks – area and perimeter comparison – each experimental condition, congruent and incongruent, was represented in total by 32 trials for the practice phase (8 × 2 blocks, filled or framed × 2 tasks – area and perimeter) and 128 trials for the proficiency phase (32 × 2 blocks, filled or framed × 2 tasks – area and perimeter). For illustration of the experimental blocks see **Figure 2**.

### Procedure

Participants were seated about 60 cm from the computer screen. The participants’ goal was to decide, in two separate tasks, which polygon has the largest area (area task) or perimeter (perimeter task). Then, they were asked to convey their answer as quickly and accurately as possible by pressing one of three marked keys on a response box (right key for right polygon, left key for left polygon, central key for equal area or perimeter). Each participant was presented with four blocks (two area blocks – filled and framed, and two perimeter blocks – filled and framed). Half the participants began with the area task (25% of participants began with a filled figures block and the rest with a framed figures block) and half with the perimeter task (25% began with a filled figures block and the rest with a framed figures block). Tasks and block performance order were counter balanced and were determined in advance according to participants’ serial numbers.

The experiment included three breaks in each block, which were terminated when participants pressed a relevant key, as well as a break of a few minutes between the sections. The stimuli in each trial began with a fixation point (small white asterisk), which appeared for 300 ms and was followed by an empty black screen for 500 ms. Then the sample polygons appeared and remained in view until the participant pressed a key but no longer than 5,000 ms. The next trial began with the fixation point. Overall, the experimental tasks took about 1 h.

### Data Analysis

#### Error Rates Analysis

Mean error rates (percentage of incorrect trials) were calculated for each participant for the practice (only the first practice was calculated for participants who failed to succeed in over 80% of the practice trials) and proficiency phases separately. Then, three-way repeated measures ANOVAs were used, with group (i.e., Control or DD) as the between-subject factor, and congruency (i.e., congruent or incongruent) and task (i.e., Area or Perimeter) as within-subject factors on the error rates of practice and proficiency trials. All of the following *F*-statistics were adjusted by the Greenhouse-Geisser correction.

In order to define the triple group × task × congruency interaction, Stroop effects of the error rates were calculated (by subtracting error rates of incongruent trials from error rates of congruent trials) for each group, in Area and Perimeter tasks separately. In order to test for a continuous exposure effect, we compared the Stroop effects of practice and proficiency phases of each task for each group separately, by using paired *t*-tests.

#### Reaction Times (RT) Analysis

Reaction times analysis was similar to error rates analysis. Mean RTs (in ms) were calculated for each participant for practice and proficiency phases separately. Then, three-way repeated measures ANOVAs were used, with group as the between-subject factor and congruency and task as within-subject factors on the RTs of practice and proficiency trials. A continuous exposure effect was observed by comparing the Stroop effects of the practice and proficiency phases of each task for each group separately, using paired *t*-tests.

#### Other Visual Features

We used repeated measures ANOVAs, similar to those described above, in order to investigate whether other visual features manipulated (i.e., figure filling, protrusion, and direction – see **Figure 1**) were possible confounders of Area and Perimeter processing.

#### Gender Differences

Gender differences were tested using independent *t*-tests.

## Results

### Gender Differences

The research sample contained a larger number of females. Accordingly, we tested gender differences (mean scores and *t*-tests are presented in **Table 2**). Females and males showed similar patterns across tasks and proficiency stages. Specifically, they showed similar Stroop effects in both tasks in both error rates and speed analyses.

**Table 2:** Mean scores and gender differences in area and perimeter tasks across different exposure phases.

	Task	Females *M* (*SD*)	Males *M* (*SD*)	*t*-test	*p*-value
Stroop effects of error	Practice phase- Area	0.01 (0.06)	0.01 (0.06)	0.468	0.642
rates (in percentage)	Practice phase- Perimeter	0.08 (0.09)	0.05 (0.07)	1.179	0.244
	Proficiency phase- Area	0.02 (0.04)	0.00 (0.03)	1.35	0.183
	Proficiency phase- Perimeter	0.01 (0.03)	0.01 (0.02)	-0.619	0.539
Stroop effects of	Practice phase- Area	308.92 (217.90)	194.09 (205.24)	1.69	0.097
reaction times (in ms)	Practice phase- Perimeter	378.85 (287.00)	266.23 (272.40)	1.25	0.214
	Proficiency phase- Area	227.66 (101.57)	270.58 (201.45)	-1.04	0.300
	Proficiency phase- Perimeter	242.73 (175.33)	193.76 (85.69)	0.969	0.337

*Stroop effects = incongruent minus congruent trials. Sig. = independent *t*-test between females and males*.

### Visual Features

Several visual features were manipulated in order to track possible confounding with the experimental variables. The filling effect (filled vs. framed figures) was not significant in the practice phase, neither for error rates [*F*_(1,55)_ = 0.10, *p* = 0.746, η^2^ = 0.002] nor for RTs [*F*_(1,55)_ = 0.39, *p* = 0.533, η^2^ = 0.008]. Moreover, there was no filling effect for error rates in the proficiency phase [*F*_(1,55)_ = 0.11, *p* = 0.741, η^2^ = 0.002]. However, participants responded more slowly to framed figures (*M* = 977.95, *SD* = 229.58) than to filled figures (*M* = 919.18, *SD* = 194.74) in the proficiency phase [*F*_(1,55)_ = 6.31, *p* = 0.015, η^2^ = 0.103]. Furthermore, in this phase, a filling × congruency interaction was evident [*F*_(1,55)_ = 5.28, *p* = 0.025, η^2^ = 0.08], such that the Stroop effect (incongruent minus congruent) was significantly greater [*F*_(1,56)_ = 4.58, *p* = 0.037, η^2^ = 0.07] for framed figures (*M* = 236.15, *SD* = 133.19) than for full figures (*M* = 196.77, *SD* = 106.72).

We further analyzed the effect of continuous exposure for each filling type separately. This was done in order to figure out whether stimuli rehearsal led to changes in the automaticity of the filling effect. On the error analysis, participants made more errors (in percentage) in congruent trials than in incongruent trials in framed figures after initial exposure (*M* Stroop effect = -0.05, *SD* = 0.07) and this difference decreased after continuous exposure (*M* Stroop effect = -0.00, *SD* = 0.02) to stimuli [*t*_(56)_ = -4.06, *p* < 0.001, *d* = 1.085]. For full figures, no difference between Stroop effects after initial (*M* = -0.03, *SD* = 0.07) or continuous exposure (*M* = -0.01, *SD* = 0.04) was evident [*t*_(56)_ = -1.51, *p* = 0.137, *d* = -0.403]. Reaction times analysis show that the Stroop effects (in ms) were larger after initial exposure and decreased after continuous exposure for both framed [initial exposure: *M* = 326.46, *SD* = 201.91; continuous exposure: *M* = 243.28, *SD* = 134.87; *t*_(56)_ = 3.71, *p* = 0.001, *d* = 0.991] and full figures [initial exposure: *M* = 290.99, *SD* = 213.14; continuous exposure: *M* = 211.03, *SD* = 123.41; *t*_(56)_ = 2.86, *p* = 0.006, *d* = 0.764].

There were no other interactions involving the filling component. No significant findings were found for error rates or RTs for other visual features manipulated, namely protrusion (protruding in or out – see **Figure 1**) and vertical or horizontal direction.

### Error Rates Analysis

#### Practice Phase

The current analysis aims to define whether Area interferes with Perimeter processing and vice versa, among participants with intact and deficient numerical processing after initial exposure to non-familiar stimuli.

Mean error rates (in percentage) in area and perimeter tasks across different exposure phases of the two groups are presented in **Table 3**. Results revealed a significant effect of group [*F*_(1,55)_ = 5.49, *p* = 0.023, η^2^ = 0.091], indicating that the DD group made more errors than the Control group. The main effect of congruency was also significant [*F*_(1,55)_ = 39.81, *p* < 0.001, η^2^ = 0.420], as congruent trials had smaller error rates than incongruent trials. No main effect of task was evident [*F*_(1,55)_ = 3.56, *p* = 0.064, η^2^ = 0.061], with similar error rates for both Area and Perimeter tasks.

**Table 3:** Mean error rates (in percentage) in area and perimeter tasks across different exposure phases of the two groups.

Group	Practice phase				Proficiency phase			
	Area		Perimeter		Area		Perimeter	
	Congruent *M* (*SD*)	Incongruent *M* (*SD*)	Congruent *M* (*SD*)	Incongruent *M* (*SD*)	Congruent *M* (*SD*)	Incongruent *M* (*SD*)	Congruent *M* (*SD*)	Incongruent *M* (*SD*)
Control	0.02 (0.03)	0.04 (0.05)	0.01 (0.02)	0.07 (0.07)	0.03 (0.03)	0.04 (0.03)	0.02 (0.02)	0.03 (0.03)
DD	0.05 (0.06)	0.05 (0.05)	0.01 (0.02)	0.12 (0.09)	0.03 (0.01)	0.04 (0.02)	0.01 (0.01)	0.03 (0.04)

However, the task × congruency interaction was significant [*F*_(1,55)_ = 22.72, *p* < 0.001, η^2^ = 0.292]. Further analysis revealed that incongruent trials were less accurate than congruent trials on the Perimeter task [*t*_(56)_ = 6.7, *p* < 0.001, *d* = 1.79]. No difference between error rates of congruent and incongruent trials was evident in the Area task [*t*_(56)_ = 1.32, *p* = 0.192, *d* = 0.352]. Importantly, no order effect was evident, as participants who started with the Perimeter task showed similar Stroop effects as participants starting with the Area task in both tasks [Area task: *t*_(55)_ = 0.767, *p* = 0.446, *d* = 0.206; Perimeter task: *t*_(55)_ = 0.004, *p* = 0.997, *d* = 0.001].

**FIGURE 3:**
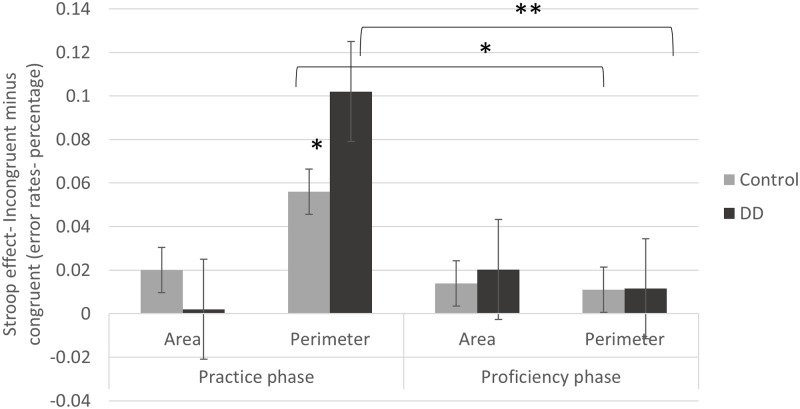
Stroop effects (incongruent minus congruent trials) on error rates (in percentage) of practice and proficiency phases in both groups (DD and Control). Error bars depict ±1 SE of the mean. Significance values: ^∗^*p* < 0.05, ^∗∗^*p* < 0.01.

Interestingly, and with high relevance to the current research questions, a triple interaction was evident [*F*_(1,55)_ = 6.69, *p* = 0.012, η^2^ = 0.109], as described in **Figure 3**. Further analysis of this interaction revealed significant effects for group [*F*_(1,55)_ = 4.73, *p* = 0.034, η^2^ = 0.079] and congruency [*F*_(1,55)_ = 47.72, *p* < 0.001, η^2^ = 0.454] in the Perimeter task (in which Area is irrelevant to the task). Moreover, in this Perimeter task, the group × congruency interaction reached significance as well [*F*_(1,55)_ = 3.81, *p* = 0.050, η^2^ = 0.065]. The Stroop effect (incongruent minus congruent) of error rates was larger for the DD group (*M* = 0.10, *SD* = 0.09) than for the Control group (*M* = 0.05, *SD* = 0.08). In the Area task, no main effect of group [*F*_(1,55)_ = 1.87, *p* = 0.176, η^2^ = 0.033] or congruency [*F*_(1,55)_ = 2.04, *p* = 0.158., η^2^ = 0.036] was evident, nor group × congruency interaction [*F*_(1,55)_ = 2.8, *p* = 0.098, η^2^ = 0.049]. In other words, in the Area task, there was no meaningful Stroop effect of error rates, and this pattern was similar for both Controls (*M* = 0.02, *SD* = 0.06) and DD (*M* = 0.00, *SD* = 0.06). In conclusion, while the DD group showed larger Stroop effects in the perimeter task, no group difference was evident in the area task.

#### Proficiency Phase

Contrary to the former analysis, this analysis aims to find out whether magnitude interference and group differences are evident after continuous exposure, when participants are well familiar with the stimuli.

The main effect of task [*F*_(1,55)_ = 5.80, *p* = 0.019, η^2^ = 0.096] was minor but significant. Importantly, no order effect was evident, as participants who started with the Perimeter task showed similar Stroop effects as participants starting with the Area task in both tasks [Area task: *t*_(55)_ = 1.3, *p* = 0.196, *d* = 0.351; Perimeter task: *t*_(55)_ = -0.55, *p* = 0.582, *d* = -0.148]. The main effect of congruency reached significance as well [*F*_(1,55)_ = 18.63, *p* < 0.001, η^2^ = 0.253]. However, no main effect of group was evident [*F*_(1,55)_ = 0.03, *p* = 0.852, η^2^ = 0.001], nor any interaction. This suggests that there was a typical Stroop effect, which was similar in pattern across groups (DD and Controls) and tasks (Area and Perimeter).

#### Continuous Exposure Effect (i.e., The Statistical Differences between Stroop Effects in the Practice vs. Task Phases)

The effect of exposure was observed by comparing the Stroop effects in the practice and proficiency phases of each task for each group separately, using paired *t*-tests. This in order to figure out whether stimuli rehearsal led to changes in automaticity of one or both magnitudes among different participants.

As described in **Figure 3**, in the Perimeter task a significant difference was found between the Stroop effects of the practice and proficiency phases for the DD group [*t*_(29)_ = 5.37, *p* < 0.001, *d* = 1.994; practice phase: *M* = 0.10, *SD* = 0.09; proficiency phase: *M* = 0.015, *SD* = 0.02]. A similar significant difference was evident for the Control group [*t*_(26)_ = 2.62, *p* = 0.015, *d* = 1.027; practice phase: *M* = 0.05, *SD* = 0.08; proficiency phase: *M* = 0.01, *SD* = 0.03]. Accordingly, the initial Stroop effects declined after practice and this pattern was evident in both groups. No difference was evident in the Stroop effects in the Area task neither for the DD group [*t*_(29)_ = -1.57, *d* = -0.583, *p* = 0.126; practice phase: *M* = 0.002, *SD* = 0.06; proficiency phase: *M* = 0.02, *SD* = 0.04] nor for the Control group [*t*_(26)_ = 1.03, *p* = 0.309, *d* = 0.403.; practice phase: *M* = 0.02, *SD* = 0.06; proficiency phase: *M* = 0.01, *SD* = 0.04].

### Reaction Time Analysis (RT)

#### Practice Phase

Mean reaction times (in ms) in area and perimeter tasks across different exposure phases of the two groups are presented in **Table 4**. When testing whether Area interferes with Perimeter processing and vice versa among participants with different numerical abilities after initial exposure to non-familiar stimuli, a main effect of congruency was found [*F*_(1,55)_ = 215.54, *p* < 0.001, η^2^ = 0.797]. Accordingly, participants responded more slowly to incongruent trials than to congruent trials. The main effect of group was not significant [*F*_(1,55)_ = 1.74, *p* = 0.191, η^2^ = 0.031], nor was the main effect of task [*F*_(1,55)_ = 2.17, *p* = 0.146, η^2^ = 0.038].

**Table 4:** Mean reaction times (in ms) in area and perimeter tasks across different exposure phases of the two groups.

Group	Practice phase				Proficiency phase			
	Area		Perimeter		Area		Perimeter	
	Congruent *M* (*SD*)	Incongruent *M*(*SD*)	Congruent *M* (*SD*)	Incongruent *M* (*SD*)	Congruent *M* (*SD*)	Incongruent *M* (*SD*)	Congruent *M* (*SD*)	Incongruent *M* (*SD*)
Control	1087.50 (246.43)	1344.09 (213.55)	1144.53 (273.77)	1421.73 (396.78)	787.55 (135.92)	995.53 (200.28)	812.73 (137.41)	1008.33 (269.53)
DD	1153.47 (289.83)	1459.74 (267.62)	1142.79 (296.73)	1564.34 (366.68)	898.98 (181.51)	1162.95 (238.72)	896.42 (249.88)	1160.36 (281.49)

However, and following the experimental hypothesis, the group × congruency interaction was found to be significant [*F*_(1,55)_ = 5.09, *p* = 0.028, η^2^ = 0.085]. The Stroop effect (incongruent minus congruent) was significantly larger [*F*_(1,55)_ = 2.25, *p* = 0.028] for the DD group (*M* = 363.9, *SD* = 147.86) than for the Control group (*M* = 266.89, *SD* = 176.33).

**FIGURE 4:**
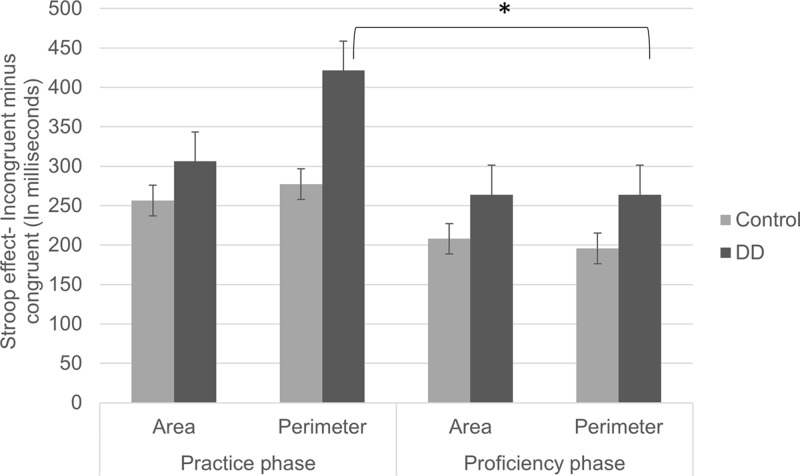
Stroop effects (incongruent minus congruent trials) on RTs of practice and proficiency phases in both groups (DD and Control). Error bars depict ±1 SE of the mean. Significance values: ^∗^*p* < 0.05.

No triple interaction was found [*F*_(1,55)_ = 0.87, *p* = 0.355, η^2^ = 0.016]. However, following scientific background and interest, we further analyzed the triple interaction (see **Figure 4**). In the Perimeter task, a significant congruency effect was found [*F*_(1,55)_ = 89.53*, p* < 0.001, η^2^ = 0.619], but no group effect [*F*_(1,55)_ = 0.74, *p* = 0.391, η^2^ = 0.013]. Furthermore, a marginally significant group × congruency interaction was evident [*F*_(1,55)_ = 3.82, *p* = 0.056, η^2^ = 0.065]. The Stroop effect (incongruent minus congruent) tended to be larger in the DD group (*M* = 421.54, *SD* = 281.00) than in the Control group (*M* = 277.19, *SD* = 275.51). In the Area task, a significant congruency was found [*F*_(1,55)_ = 93.63, *P* < 0.001, η^2^ = 0.630], but no group effect [*F*_(1,55)_ = 2.16, *p* = 0.147, η^2^ = 0.038]. No group × congruency interaction was found [*F*_(1,55)_ = 0.72, *p* = 0.397, η^2^ = 0.013]. In other words, in the Area task, there was a typical Stroop effect, and this pattern was similar for Controls (*M* = 256.59, *SD* = 198.83) and DD (*M* = 306.27, *SD* = 236.10). Similar to error rates analysis, the DD group tended to show larger Stroop effects (marginally significant) in the perimeter but not in the area task.

#### Proficiency Phase

While aiming to find out whether magnitude interference and group differences were evident after continuous exposure, results revealed a main effect of group [*F*_(1,55)_ = 6.28, *p* = 0.015, η^2^ = 0.103]. DDs’ RTs were significantly slower than Controls’. The main effect of congruency was also significant [*F*_(1,55)_ = 241.8, *p* < 0.001, η^2^ = 0.815], such that participants responded more slowly to incongruent trials than to congruent trials. However, the main effect of task did not reach significance [*F*_(1,55)_ = 0.16, *p* = 0.684, η^2^ = 0.003], such that participants responded similarly to Area and to Perimeter tasks.

Importantly and following the experimental hypothesis, the group × congruency interaction was found to be significant [*F*_(1,55)_ = 04.30, *p* = 0.043, η^2^ = 0.073]. The Stroop effect (incongruent minus congruent) was significantly larger [*F*_(1,55)_ = 4.01, *p* = 0.043, η^2^ = 0.068] for the DD group (*M* = 263.95, *SD* = 107.74) than for the Control group (*M* = 201.79, *SD* = 118.39).

No triple interaction was found between group, congruency, and task in the proficiency phase [*F*_(1,55)_ = 0.06, *p* = 0.794, η^2^ = 0.001]. However, following scientific interest, the double interaction between congruency and group was analyzed separately in each task. In the Perimeter task, significant congruency [*F*_(1,55)_ = 120.68, *p* < 0.001, η^2^ = 0.687] and a marginally significant group effect [*F*_(1,55)_ = 3.74, *p* = 0.058, η^2^ = 0.064] were found. However, no group × congruency interaction was evident [*F*_(1,55)_ = 2.66, *p* = 0.108, η^2^ = 0.046] and both groups showed typical Stroop effects (DD: *M* = 263.93, *SD* = 150.83; Control: *M* = 195.60, *SD* = 165.02). Similarly, in the Area task, significant congruency [*F*_(1,55)_ = 192.45, *p* < 0.001, η^2^ = 0.778] and group effects [*F*_(1,55)_ = 8.25, *p* = 0.006, η^2^ = 0.131] were found. However, no group × congruency interaction was found [*F*_(1,55)_ = 2.7, *p* = 0.106, η^2^ = 0.047] and both groups showed typical Stroop effects (DD: *M* = 263.97, *SD* = 144.12; Control: *M* = 207.98, *SD* = 107.81). Accordingly, after continuous exposure to stimuli, both groups showed typical Stroop effects in both tasks.

#### Continuous Exposure Effect (i.e., The Statistical Differences between Stroop Effects in the Practice vs. Task Phases)

We analyzed the effect of exposure separately for each group in each task. This was done in order to figure out whether stimuli rehearsal led to changes in automaticity of one or both magnitudes among different participants. In the Perimeter task (in which Area is irrelevant to the task), a significant difference was found in the Stroop effects (congruent minus incongruent) between practice and proficiency phases for the DD group [*t*_(29)_ = 3.64, *p* = 0.001, *d* = 1.351]. Accordingly, high initial Stroop effects (*M* = 421.54, *SD* = 281.006) declined after practice (*M* = 263.93, *SD* = 150.83). No significant difference was evident between Stroop effects in the practice and proficiency phases for the Control group [*t*_(26)_ = 1.62, *p* = 0.115, *d* = 0.635; practice phase: *M* = 277.19, *SD* = 275.51; proficiency phase: *M* = 195.60, *SD* = 165.02]. No difference in Stroop effects was evident in the Area task (in which Perimeter was irrelevant to the task), neither for the DD group [*t*_(29)_ = 1.17, *p* = 0.248, *d* = 0.434; Practice phase: *M* = 306.27, *SD* = 236.10; Proficiency phase: *M* = 263.97, *SD* = 144.12], nor for the Control group [*t*_(26)_ = 1.31, *p* = 0.202, *d* = 0.513; practice phase: *M* = 256.59, *SD* = 198.83; proficiency phase: *M* = 207.98, *SD* = 107.87].

## Discussion

The present study investigated the automaticity of area and perimeter processing at different exposure levels in adults with deficient and intact numerical abilities.

To the best of our knowledge, this is the first study to show that individuals with DD process area and perimeter information differently and in a less automatic manner than peers with intact numerical competence. After initial exposure to stimuli, area processing was more automatic than perimeter processing for both groups, as represented by task × congruency interaction, evident in error rate analysis. Specifically, significant Stroop effects (slower responses to incongruent vs. congruent trials) were evident in perimeter and not in area tasks. However, this pattern was more prominent among the DD group, appearing in both error rates and speed analyses, implying a magnitude processing deficit. Together with previous evidence (Babai et al., 2006), the current findings show that area interferes with perimeter processing but not vice versa. This pattern suggests that while area processing may be innate, perimeter processing is acquired. After continuous exposure, the difference between area and perimeter was no longer evident. Furthermore, significant Stroop effects (in speed analysis) show that both magnitudes were automatically processed and interfered with each other to a similar degree in both groups. However, we found that domain general learning and inhibition deficits are also involved in DD. Specifically, we found firm group × congruency interactions in the speed dimension. Namely, the DD group showed larger Stroop effects, which were evident across all exposure levels. Overall, the findings imply that deficient performance of participants with DD may not be restricted to numerical processing.

### Automatic Processing of Area and Perimeter

In the current work, area and perimeter were both found to be automatically processed, as shown by the existence of significant Stroop effects (slower responses to incongruent vs. congruent trials), evident from speed analysis in both area and perimeter tasks. In other words, the irrelevant aspect (area or perimeter) was involuntarily processed. As in the case of the numerical Stroop (Henik and Tzelgov, 1982), bidirectional effects were evident between the two magnitudes. The fact that both aspects are automatically processed is compatible with recent research highlighting the existence of a general magnitude processing mechanism (Gebuis and Reynvoet, 2012b; Newcombe, 2014; Leibovich et al., 2016).

Our findings are partially consistent with previous studies (Babai et al., 2006) showing that the area aspect interferes with perimeter judging but not vice versa. In the current study, perimeter processing seems to be automatic as well and to interfere with area processing after continuous exposure to the stimuli. However, some of our findings support the assumption that the area aspect is more perceptually salient. In the analysis of error rates in the practice phase we found Stroop effects (when congruent trials were more accurate than incongruent trials) in the perimeter but not in the area task. Area saliency probably relates to the fact that it occupies a larger space (in square meters) (Abbott, 1976). Moreover, magnitude saliency can differ due to task demands (Spelke et al., 2010). From this perspective, the processing of surface layouts (perimeter), which is crucial for navigation tasks (Cheng and Newcombe, 2005), is probably less vital than the figures’ area in order to decide which small-scale 2D figure (Lee and Spelke, 2011) is larger, as required in the current task.

Since area saliency disappeared in the proficiency phase, we can assume that exposure levels also account for magnitude saliency. Accordingly, after practicing, participants were able to successfully inhibit irrelevant salient area magnitude information, and no difference was found between area and perimeter Stroop effects. Based on previous study that found that as few as eight rehearsals trials, contributed to changes in brain functions (Ischebeck et al., 2007), we may argue, that in the current study which included larger number of stimuli, a meaningful learning indeed occurred. The findings are consistent with intervention studies indicating that attracting attention to the perimeter aspect (either by adding warnings or by presenting a polygon’s perimeter in discrete units) improved participants’ accuracy rates in similar geometric tasks (Babai et al., 2015, 2016). One should notice that the current research did not include direct comparison between numerical and continuous magnitudes. Accordingly, no conclusions regarding the interactions between area, perimeter, and numeracy in numerical tasks, such as dot arrays judgment tasks (as in Gebuis and Reynvoet, 2012a,b), can be made.

### DD and Automatic Processing of Magnitudes

When faced with unfamiliar stimuli in the practice phase, a triple group × task × congruency interaction was evident in error rates analysis. Participants with DD showed larger Stroop effects compared to the control group in the perimeter task but not in the area task (as in Babai et al., 2006). This pattern was also marginally significant for speed analysis. These findings indicate that individuals with deficient numerical processing also struggle with magnitude, non-numerical processing (as suggested by Leibovich et al., 2016). Moreover, group differences were significant when trying to ignore the irrelevant but salient area magnitude (Babai et al., 2006; Stavy et al., 2006; Stavy and Babai, 2008, 2010). Accordingly, the findings suggest that individuals with DD are deficient not only in the processing of numerical stimuli (e.g., Kaufmann and von Aster, 2012), but also in the processing of continuous magnitudes (in this specific case, perimeter).

Nevertheless, with practice and growing proficiency in the tasks’ demands, no triple interactions were evident. The difference between perimeter and area tasks disappeared and both groups showed typical Stroop effects for both magnitudes. In other words, both magnitudes were processed automatically. Accordingly, group differences in magnitude comparison tasks may vary due to task familiarity or proficiency levels. Developmental studies regarding the numeric Stroop task indicate that one magnitude (physical size) seems to be more salient and to interfere with the other (symbolic numbers) among first graders who have no formal numerical education. With educational progress and repeated experience with numbers, both magnitudes are automatically processed and bidirectional effects are evident (Girelli et al., 2000; Rubinsten et al., 2002). Here we show a similar pattern, as task practice resulted in bidirectional effects for both groups and both magnitudes interfered with each other’s judgment.

Further analysis revealed that the reduction of Stroop effects in the perimeter task was more prominent for the DD group. Accordingly, participants with DD showed automatic processing of the perimeter aspect only after continuous exposure to stimuli. Namely, they had to intentionally learn to process the perimeter aspect. The findings emphasize the need to summon intensive exposure to magnitude, as well as to numerical information, in order to enhance compensation of DDs’ core deficits (e.g., Kaufmann et al., 2003; Wilson et al., 2006).

Beyond the earlier discussion regarding magnitude saliency, a group × congruency interaction was evident on the speed dimension across all exposure phases. Participants with DD systematically struggled to inhibit irrelevant magnitude information, manifested by larger Stroop effects, regardless of task type – area or perimeter. This pattern is compatible with inhibition deficits often associated with DD (Wang et al., 2012; Szucs et al., 2013a; Bugden and Ansari, 2015). As suggested by Aubin et al. (2016), individuals with DD might fail to consolidate magnitude knowledge and to produce the shift from the “slow” computing neural network to the “fast” retrieval network. Hence, they must invest more cognitive efforts in order to ignore irrelevant magnitude information. According to the magnitude mechanism hypothesis, a separate and more precise representation of each magnitude dimension occurs across development and becomes stronger with formal education (Newcombe, 2014). In line with this theory, people with DD struggle to effectively process different magnitude aspects of the stimulus in order to extract the proper magnitude on one hand and ignore the irrelevant magnitude on the other (Gebuis and Gevers, 2011). Hence, individuals with DD may show a developmental gap (Rubinsten and Henik, 2005), demonstrated by a difficulty in differentiating between magnitudes. Based on the current findings, we cannot specify whether individuals with DD have a deficient, domain-general inhibition mechanism (Soltész et al., 2007), or whether these inhibition deficits are exclusive to magnitude processing (De Visscher and Noël, 2014). It is also plausible that multiple neuro-cognitive components, domain-specific and domain-general, account for DD (Rubinsten and Henik, 2009; McCaskey et al., 2017). For instance, some researchers argue that DD relates to visual-spatial deficits (Ashkenazi et al., 2013; Szucs et al., 2013a; Bugden and Ansari, 2015). The fact that the DD group performed worse than the control group on reading tasks may strengthen this argument, as the reading process is known to involve visual-spatial processing (Cohen et al., 2000; Facoetti et al., 2000).

Since assessment tools and inclusion criteria vary between studies concerning numerical difficulties (Kaufmann et al., 2013), the debate on which domain-specific and domain-general deficits underlie numerical difficulties has not yet been fully addressed. There is also a need to develop a massive body of research involving developmental research and using other continuous magnitude assessment tasks. However, it is worth noting that, based on developmental research, Leibovich and Henik (2014), Leibovich et al. (2016) suggest a developmental model of the magnitude processing system: at the first step infants process mostly continuous magnitudes (for example, Clearfield and Mix, 1999). With the developmental process, they learn about the correlation between continuous and discrete magnitudes (numbers) and are subsequently able to process both types of magnitude separately (Leibovich and Henik, 2014). Development of cognitive control, including inhibition mechanisms (Davidson et al., 2006), is crucial in order to enable differentiation between incongruent magnitudes (Leibovich et al., 2016). The proposed model has not yet been systematically validated, and the study of possible developmental gaps that might shed light on how numerical difficulties arise and develop, has not been established. However, the current study implies that DD deficits indeed relate to a failure to differentiate between magnitudes and to inhibit the irrelevant magnitude.

### Other Visual Properties

The uniqueness of the current Stroop–like task enabled us to look at the automaticity of framed vs. filled figures separately. Results show that participants showed larger Stroop effects in framed vs. filled trials, regardless of trial type (area or perimeter) and group (DD or control). These findings are not consistent with previous ones suggesting that framed figures emphasize the perimeter aspect and thus help ignore irrelevant area information (Stavy and Babai, 2008). Magnitudes tend to become confounded in a natural environment (Gebuis and Reynvoet, 2012a,b). Accordingly, it is possible that filled figures, occupying as they do larger magnitudes than framed figures, increased area and perimeter’s natural confound.

Additional visual variables were examined in order to eliminate possible alternative explanations of the results. While no full control of other visual features is possible (Gebuis and Reynvoet, 2012a,b), changes in these variables appear to be good indicators that participants’ performance was affected by the manipulation of experimental variables (i.e., area and perimeter). No group differences were found in any of the other visual features, including protrusion and vertical or horizontal direction. Hence, we can argue that group differences arise from magnitude processing rather than from interference of irrelevant visual features.

### Limitations

One limitation of the current study is that all participants were well-educated students and hence might not be representative of the entire DD demographic. Furthermore, the relationship between magnitude and numerical processing was not directly assessed in the current research, a fact that reduces our ability to generate firm conclusions about the role of magnitude processing in numerical processing.

## Conclusion and Implications

The current study indicates that both area and perimeter magnitudes are automatically processed and that participants with DD find it harder to ignore irrelevant magnitude information, especially salient area information. We assume that our findings derive from an inhibition deficit related to magnitude processing (Wang et al., 2012; De Visscher and Noël, 2014). The findings are also compatible with recent theories regarding the general magnitude processing mechanism (Leibovich and Henik, 2014; Newcombe, 2014; Lourenco and Bonny, 2016; Leibovich et al., 2016). On the other hand, we show that continuous exposure to stimuli was effective and resulted in similar patterns, namely typical Stroop effects for both magnitudes, for both groups. This fact is important for the planning of future intervention programs emphasizing the vitality of massive exposure to magnitude related stimuli in order to overcome the core deficits related to DD.

One main conclusion that can be deduced from the above is the need to develop other tasks assessing non-numerical magnitude processing, such as the task presented in the current work. Investigating multiple magnitude processing is crucial for both research and educational fields. On the one hand, there is a need for better knowledge about how multiple magnitude processing develops and occurs on the neuronal and behavioral levels in order to develop adaptive behavior. Traditionally, research methods have aimed to assess numerical processing exclusively and to block interfering magnitude information. On the other hand, the current and recent works (Gebuis and Reynvoet, 2012b; Szűcs et al., 2013b; Newcombe, 2014) stress that numerical and magnitude confounding should receive more attention in order to understand intact and impaired numerical processing. Furthermore, since non-numerical magnitude processing is crucial for higher education in science and mathematics (Wai et al., 2009; Marghetis et al., 2016), it is necessary to relate to non-numerical magnitude processing in educational research and in math curricula.

## Author Contributions

HE-L and OR contributed significantly to research design, data interpretation, and manuscript drafting. HE-L collected the data.

## Conflict of Interest Statement

The authors declare that the research was conducted in the absence of any commercial or financial relationships that could be construed as a potential conflict of interest.
